# Using Behavioral Consensus to Learn about Social Conventions in Early Childhood

**DOI:** 10.3389/fpsyg.2016.01510

**Published:** 2016-10-05

**Authors:** Wanying Zhao, Andrew S. Baron, J. K. Hamlin

**Affiliations:** Department of Psychology, University of British ColumbiaVancouver, BC, Canada

**Keywords:** social conventions, selective learning, social groups, social evaluation, innovation, cultural learning

## Abstract

Adults make inferences about the conventionality of others’ behaviors based on their prevalence across individuals. Here, we look at whether children use behavioral consensus as a cue to conventionality, and whether this informs which cultural models children choose to learn from. We find that 2- to 5-year old children exhibit increasing sensitivity to behavioral consensus with age, suggesting that like adults, young humans use behavioral consensus to identify social conventions. However, unlike previous studies showing children’s tendencies to prefer and to learn from members of a consensus, the present study suggests that there are contexts in which children prefer and learn from unconventional individuals. The implications of these different preferences are discussed.

## Introduction

Humans are creatures of social convention. Social conventions prescribe group-specific ways of being, facilitating smooth, and cooperative social interactions amongst group members (Cialdini and Trost, 1998; Henrich and Henrich, 2007). They serve as symbolic markers of group membership, making it easy to identify whether an individual belongs to an in-group or to an out-group (Boyd and Richerson, 1987; Fitch, 2000). Conventional behaviors are often also of high quality, because they have been vetted by numerous people over repeated use, and therefore represent an efficient and effective way of doing things. Social conventions shape the ways in which we fulfill our most basic biological functions, including how we eat, sleep, and have sex (e.g., Ford and Beach, 1951; Jenni and O’Connor, 2005; Schultz et al., 2007). Members of every culture follow their societies’ rules for how to behave from early in life (Henrich et al., 2001; Killen and Smetana, 2005), and are exquisitely sensitive to whether others follow group conventions, willingly punishing unconventional behaviors at personal cost (Gintis, 2000; Fehr et al., 2002; Henrich, 2006). Indeed, even very young children rapidly acquire new social rules, and protest if those rules are violated (Schmidt et al., 2010; Schmidt and Tomasello, 2012). Here, we explore the development of sensitivity to social convention by examining whether young children exhibit social preferences for individuals who adhere to a group’s shared behavior (e.g., a dance), and whether these preferences influence children’s selection of whom to learn from.

Adults identify potential social conventions by looking to the behaviors of the majority, and, once a convention is identified, modify their behaviors to reflect it (Latané and Darley, 1968; Prentice and Miller, 1993; Cialdini et al., 2006; Goldstein et al., 2008). A growing body of recent work suggests that young children are similarly sensitive to the behaviors of the majority, and readily use majority behaviors to learn about their culture. For example, when presented with several potential informants, 3- and 4-year-olds preferentially accept information from a 3-member consensus rather than a lone individual (Corriveau et al., 2009); children’s tendency to follow the majority is so strong that it can even lead children to discount their own perceptual judgments (Corriveau and Harris, 2010; see Asch, 1952 for adult evidence). Selectively learning from those who produce familiar conventional behaviors is already observable in infancy: 14-month-olds are more likely to imitate individuals who have produced conventional versus unconventional acts (e.g., putting shoes on one’s feet versus one’s hands; Zmyj et al., 2010). Finally, if no consensus information is currently observable, young children readily use indirect cues to majority behavior: 3-year-olds preferentially learn from familiar models versus unfamiliar ones (Reyes-Jaquez and Echols, 2013), and 14-month-olds are more likely to imitate in-group versus out-group members (Buttelmann et al., 2013). Together, these findings suggest that young children are sensitive to potential sources of conventional knowledge, and that they selectively take on new information from these sources (Bar-Haim et al., 2006; Kinzler et al., 2007; Powell and Spelke, 2013).

While it is often beneficial to follow conventions performed by the majority of group members, there may be situations in which doing so is less optimal. For instance, sometimes the majority is simply incorrect, and so viewing majority behaviors in some privileged light would lead to error (e.g., Prentice and Miller, 1993). Indeed, despite work demonstrating that children sometimes slavishly follow the majority (Corriveau and Harris, 2010), other studies suggest that children are sensitive to the possibility that majorities can be wrong. For example, Schillaci and Kelemen (2014) found that 4-year-old children followed the consensus when majority and minority opinions were equally likely to be true; however, children followed a minority opinion if the minority opinion were more plausible. In a related study, 4- and 5-year olds were equally likely to learn about how to open novel puzzle boxes from an individual versus a group when opening success-rates were equated; however, children were more likely to learn from a successful individual than from an unsuccessful group (Scofield et al., 2013; Wilks et al., 2015). Together, these studies suggest that children’s sensitivity to majority behaviors is flexible: they will avoid learning from the majority when the majority is clearly unsuccessful.

Of course, young learners will frequently be confronted with situations in which it is impossible to determine the relative “success” of a given behavior, given that much of what humans do is causally opaque. For example, in many language learning situations, all labels are unfamiliar to the learner, and there is no way of determining from the input which labels go with which concepts. In addition, there are entire classes of human behaviors, for example dances and rituals, which are causally opaque and socially motivated, and thus have no physically evaluable outcomes (Legare et al., 2015). The learning of rituals requires conforming to the way group members perform actions with a high degree of accuracy (Herrmann et al., 2013; Watson-Jones et al., 2014). Presumably, in these situations children should be particularly motivated to acquire the behaviors of the majority, and to learn further information from those who have produced majority behaviors. However, although to date much research has established that children preferentially accept novel labels or artifact functions from a majority (Corriveau and Harris, 2010; Chen et al., 2013; Schillaci and Kelemen, 2014), to our knowledge, few studies have yet explored whether children are sensitive to group consensus in arbitrary action domains like dancing (for discussion see, Legare and Nielsen, 2015). The current studies were designed to fill this gap in the literature, by examining children’s reactions to and preferential learning from an individual who performs the same-dance as several other individuals, versus an individual who performs a novel-dance. We hypothesized that children would identify the dance as a convention or a ritual behavior, and would therefore prefer and preferentially learn from individuals who perform it.

## The Present Study

Children watched a live action dance show, depicting generic Smurf plush toys. Four identical Smurfs performed sequences of arbitrary physical movements making up different dances. The experiment was conducted following the recent release of a Smurfs movie, so the toys were familiar and engaging to many children. Smurfs look like members of a distinct social group, and were introduced as such by the Experimenter, by saying “Do you know who these guys are? They are Smurfs!.”

We wished to know if kids prefer individuals who follow a consensus over those who do not. However, we needed to ensure that any observed preferences would in fact be due to consensus, and not due to something simpler, such as behavioral familiarity or exposure frequency. To address whether children differentiate between group-relevant conventions (behaviors that are performed by multiple different individuals in a group) and simple behavioral familiarity (behaviors that are performed frequently), participants were randomly assigned to either the “Consensus condition” or the “Repetition condition.” In the Consensus condition, children were introduced to the group of Smurfs and then viewed four Smurfs (heretofore the Demonstrators) perform the very same-dance, one at a time, for a total of four dances. In the Repetition condition, children were introduced to the same four Smurf Demonstrators, but then viewed just one Demonstrator perform the same-dance repeatedly, for a total of four dances. Following the Demonstrator(s)’ dances, one new Smurf performed the dance that the Demonstrator(s) had just performed (heretofore the “same-dance” Protagonist), and a second new Smurf Protagonist performed a novel-dance (the “novel-dance” Protagonist).

Subsequently, we explored children’s social preferences for and learning tendencies from the novel- and same-dance Protagonists. To measure social preference, children were presented with the two Protagonists and asked to identify which they liked. To measure learning, each Protagonist provided a label for a novel object, and children were asked to endorse one label or the other. We reasoned that if children form preferences and selectively learn based on conventionality, they should distinguish the same-dance from the novel-dance Protagonists in the Consensus condition but not the Repetition condition. However, if children prefer individuals simply based on behavioral familiarity, then they should show similar preferences and learning in both the Consensus and Repetition conditions. Furthermore, if children deem conventional knowledge to be beneficial, they should select and learn from the same-dance Protagonist.

### Methods

#### Participants

One hundred and ninety-eight children participated in the study (Mean age = 3.98, 44.6% female, range = 2 years, 0 days – 6 years, 0 days, with an equal number of children above and below the mean age). Data from 19 children were excluded due to parental interference, or to providing no choice on both the dependent measures. Participants were recruited during a visit to the Living Lab at Science World, a local Science Centre, and tested in a sound proof room dedicated for behavioral science research. A legal guardian provided consent for child participants. The majority of participants were White and all were English speaking (though not necessarily as a first language), though a range of ethnicities and SES backgrounds were represented.

#### Procedures

##### Introduction

**FIGURE 1:**
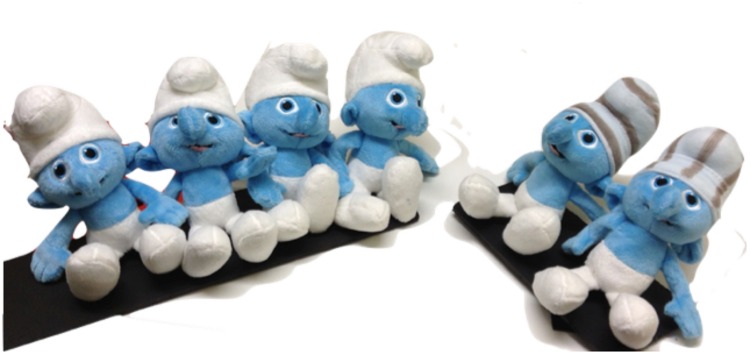
**Smurf puppets used in Study 3: demonstrators on the left, and Protagonists on the right**.

Children were tested individually in a testing room, seated across a table from the Experimenter. To introduce the study, the Experimenter gestured to four Demonstrator Smurfs seated in a group to the left and two Protagonist Smurfs seated in a group to the right, all across from participants on the table, and asked, “Do you know who these guys are? That’s right, they’re Smurfs! We’re going to see these Smurfs do a dance today.” Protagonists were distinguishable from each other by wearing vertically striped vs. horizontally striped hats; they were otherwise identical (**Figure 1** for stimuli). After the introductions, Protagonists were removed from the table and placed out of sight, while Demonstrators remained seated on the table.

##### Demonstration

Each dance began with a Demonstrator saying “My turn!” in an excited voice, and then moving to the center of the stage. The Demonstrator then performed either the “Jumping” dance or the “Swaying” dance (counterbalanced across participants). The Jumping dance consisted of jumping up and down four times, and the Swaying dance consisted of swaying side to side four times; both dances were performed at the same rhythm, for the same total duration, and Smurfs moved approximately the same distance (up and down, or side to side) from their starting places during each one. After completing the dance, the Demonstrator returned to its initial position. In the Consensus condition, each of the 4 Demonstrators performed the same-dance in turn. In the Repetition condition, one of the 4 Demonstrators performed the same-dance 4 times in a row. To maximize the similarity between the Consensus and Repetition conditions, the Repetition Demonstrator said “My turn!” at the start of each dance, and returned to his original position between each dance. After the Demonstrators’ performance, the Experimenters said, “ok Bye! See you later!” and were removed together from the table.

##### Protagonist phase

Following the demonstration phase, the Protagonists were brought out to the table and reintroduced to the participant. The Experimenter said, “Let’s see what these guys do!” One of the Protagonists performed the same-dance as the Demonstrators, and the other Protagonist performed the novel-dance. For example, when the Demonstrators performed the Jumping dance, the same-dance Protagonist also performed the Jumping dance, while the novel-dance Protagonist performed the Swaying dance. Dances performed by the Demonstrator(s), performance orders, and Protagonist type (whether they performed the same or novel-dance) were counterbalanced across subjects.

##### Preference

After each child viewed the dances, they were presented with the two Protagonists side-by-side in the center of the table and asked, “Which one do you like more?” If the child did not provide a choice after 3 s, they were prompted by the Experimenter, “Do you like one of these guys more than the other?” A small number of children (*n* = 9, 4.3% of the sample) claimed to like the two Protagonists equally; their responses for Liking were excluded from the analyses. Responses from 176 children were included in the analyses reported below.

##### Learning

Following their response for Liking, we examined whether children exhibit a preference for one of the two actors in a novel context probing knowledge about object labels. For this task, an unfamiliar object (a metal thermos cap) was introduced. The Experimenter held the object and rotated it in different angles, then placed it on the table in front of the child. Children were asked if they knew what it was; none did. The Experimenter then said, “These guys have different names for this object, let’s hear what they think it’s called.” The Experimenter then picked up each of the Protagonists in turn to point at the cap and label it; one said, “It’s a pavo!” and the other said, “It’s a loba!” Children were then asked, “What do you think it’s called?” Children’s responses were recorded, and all participants were thanked and given a sticker for their participation. If children’s choice of cultural models is motivated by learning from those they like, we should expect responses for this question to be correlated with their choice of Protagonist.

### Results

#### Liking

In response to the question “who do you like more?” children picked the novel-dance Protagonist more often in the Consensus condition (57 of 81, or 70.3%, binomial probability test, *p* < 0.001, two-tailed), but did not show a preference in the Repetition condition (51 of 90, or 56.6%, binomial probability test, *p* = 0.246, two-tailed). There was marginally significant effect of condition (Pearson’s χ^2^ = 3.44, *p* = 0.064). This supports our prediction that children’s social preferences are informed by what an individual does, relative to the overall distribution of observed behaviors. However, the preference for the novel-dance Protagonist opposed our predictions, and suggests that children sometimes prefer individuals who introduce novel, rather than conventional, behaviors. However, subsequent age analyses revealed that these preferences showed marked differences by age.

##### Liking by age

**FIGURE 2:**
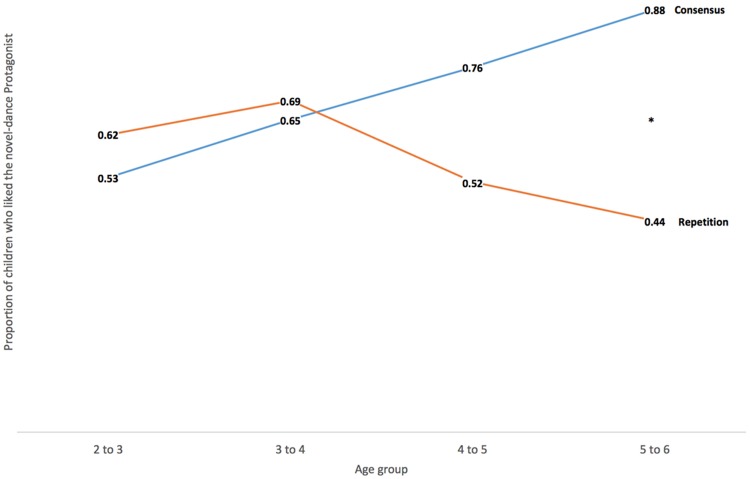
**Proportion of children who liked the novel-dance Protagonist, by age, and by condition**.^∗^*p* < 0.05.

Two- and three-year-old children did not show significant preferences for either Protagonist in either Consensus or Repetition conditions (see **Table 1** for children’s Protagonist choices by age and study condition). The proportion of 2-year-olds who preferred the novel-dance Protagonist was 53% in the Consensus condition (binomial probability test, *p* = 1), and 62% in the Repetition condition (binomial probability test, *p* = 0.27), and the proportion of 3-year-olds was 65% in the Consensus condition (binomial probability test, *p* = 0.21), and 69% in the Repetition condition (binomial probability test, *p* = 0.21). Furthermore, there was no significant difference between conditions at either age (Pearson χ^2^= 0.37, *p* = 0.543 for 2 year-olds, *N* = 46 and χ^2^= 0.05, *p* = 0.823 for 3 year olds, *N* = 40). Children started to show a significant preference for the novel-dance character at age 4 (proportion choosing novel-dance Protagonist = 76%, *p* = 0.016, two-tailed), and did so only in the Consensus, but not the Repetition, condition (proportion choosing novel-dance Protagonist = 48%, *p* = 1; Pearson χ^2^ = 3.26, *p* = 0.071, *N* = 54). This pattern became more pronounced by age 5, where 88% of children in the Consensus condition chose the novel-dance Protagonist (*p* = 0.006), compared to 44% in the Repetition condition (*p* = 0.81). The difference between conditions is significant by a Pearson χ^2^ (*p* = 0.009, *N* = 34). In summary, the overall pattern described earlier was due to both the 4 and 5 year olds differentiating between Repetition and Consensus conditions, and preferring the novel-dance Protagonist in the Consensus condition. See **Figure 2** for graph depicting the proportion of children who chose the novel-dance Protagonist.

**Table 1:** Proportion of children who liked the novel-dance Protagonist, by age and by condition.

	Consensus		Repetition		Difference between conditions	
Age group	Pr (novel)	*p*-value^∗^	Pr (novel)	*p*-value^∗^	*p*-value^∗∗^	*n*
2–3	0.53	1	0.62	0.27	0.543	46
3–4	0.65	0.21	0.69	0.21	0.823	40
4–5	0.76	0.016	0.52	1	0.071	54
5–6	0.81	0.006	0.44	0.81	0.009	34

*^∗^Binomial probability test (two-tailed), ^∗∗^Pearson χ^2^ test.*

##### Magnitude estimates for liking

To estimate the effect sizes of the comparisons discussed above, we employed a second analytic strategy to predict children’s likelihood of preferring the novel-dance Protagonist. For this analysis, a binary logistic regression was run using combined data from participants of all age groups. In the binary logistic regression model, condition (Repetition, Consensus), age (centered on sample mean of 3.98), sex (female, male), and a condition by age interaction term were entered as model predictors for likelihood of choosing the novel-dance Protagonist. An omnibus test of the model was significant (χ^2^(4) = 10.977, *p* = 0.027), improving our ability to predict infants’ Protagonist choices on 3% of cases. Together, the coefficients explained approximately 8.2% of the variance in target choice (Nagelkerke *R*^2^ = 0.082). Logistic Regression coefficients and standard errors for each predictor variable are shown in **Table 3**.

Looking at individual predictors, analyses revealed that being in the Consensus condition predicted children being 1.7 times as likely to pick the novel-dance Protagonist, compared to the Repetition condition (or 0.58 times as likely to choose the same-dance Protagonist; logistic regression coefficient = -0.546, *p* = 0.096, Odds Ratio = 0.579). Sex was also a significant predictor, such that boys were nearly twice as likely to prefer the novel-dance Protagonist as girls (or 0.51 times as likely to prefer the same-dance Protagonist; logistic regression coefficient = -0.673, *p* = 0.042, Odds Ratio = 0.51) regardless of condition. While we did not predict this difference, such a result is consistent with previous findings of gender differences in conformity (e.g., Maccoby and Jacklin, 1974; Eagly, 1978; Cooper, 1979; Eagly and Carli, 1981). Age alone was not a significant predictor; however, children’s likelihood of differentiating their choice by condition increased with age, indicating that for every 1 year increase in age, children were 1.8 times as likely to prefer the novel-dance Protagonist in the Consensus condition as compared to the Repetition condition (or 0.57 times as likely to prefer the same-dance Protagonist; logistic regression coefficient = -0.583, *p* = 0.055, Odds Ratio = 0.57).

#### Learning

**FIGURE 3:**
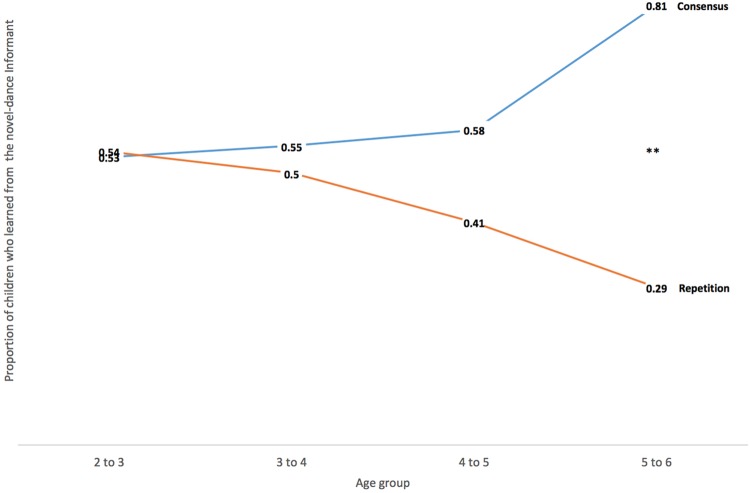
**Proportion of children who learned from the novel-dance Protagonist, by age, and by condition.** ^∗∗^*p* < 0.01.

Overall, children were more likely to adopt the label for the unfamiliar object from the novel-dance Protagonist in the Consensus condition (60.5% or 49 of 81 children), than in the Repetition condition (39 of 89, or 43.8%; Pearson χ^2^ test *p* = 0.029, two-tailed). (See **Table 2** for proportion of children who learned from the novel-dance Protagnoist, by age, and by condition). Consistent with our results for liking, children appeared sensitive to the distribution of observed behaviors for making informant choices. In particular, children adopted the unfamiliar object label from a Smurf who performed a novel-dance, after having seen a group of Smurfs first perform a shared dance. As with liking judgments, children’s informant preference became increasingly pronounced with age. See **Figure 3** for graph depicting the proportion of children who learned from the ovel-dance Protagonist.

**Table 2:** Proportion of children who learned from the novel-dance Protagonist, by age, and by condition.

Age group	Consensus		Repetition		Difference between conditions
	Pr (novel)	*p*-value^∗^	Pr (novel)	*p*-value^∗^	*p*-value^∗∗^	*n*
2–3	0.53	1	0.54	1	1	46
3–4	0.55	1	0.5	1	1	40
4–5	0.58	0.55	0.41	0.44	0.22	54
5–6	0.81	0.024	0.29	0.14	0.003	34

*^∗^Binomial probability test (two-tailed), ^∗∗^Pearson χ^2^ test.*

##### Effects of age

Two-, three-, and four-year old children were equally likely to learn from the novel-dance Protagonist as the same-dance Protagonist in both Consensus and Repetition conditions (proportion preferring to learn from the novel-dance Protagonist, at 2–3 years = 53%, *p* = 1, *N* = 46; at 3–4 years = 55%, *p* = 1, *N* = 40; at 4–5 years = 58%, *p* = 0.58, *N* = 54). Only 5-year-olds made a significantly different choice of informant in the Consensus condition than from the Repetition condition, with 81% preferring to learn from the novel-dance Protagonist in the Consensus condition (binomial probability test, *p* = 0.024, two-tailed), and 29% in the Repetition condition. The difference in choice patterns between Consensus and Repetition conditions was significant by a Pearson χ^2^ test (χ^2^= 8.93, *p* = 0.003, *N* = 34).

##### Magnitude estimates for learning

Using the same analytic approach as for the liking measure, we conducted a binary logistic regression to examine the magnitude of difference in likelihood by age and by condition. Condition, age, sex, and age-by-condition interaction term were entered as model predictors for likelihood of choosing the novel-dance Protagonist. An omnibus test of the model was significant (χ^2^(4) = 10.997, *p* = 0.027), improving our ability to predict infants’ informant choice on 9.2% of cases. Together, the coefficients explain approximately 8.1% of the variance in informant choice (Nagelkerke *R*^2^ = 0.081; see **Table 3** for binary logistic regression coefficients and standard errors for each predictor).

**Table 3:** Liking and learning from the same-dance Protagonist, predicted by age, sex, condition, and age-by-condition interaction term.

Predictors	Liking (*SE*)	Learning (*SE*)
Age (centered)	0.068 (0.184)	0.334 (0.192)^†^
Sex	-0.673 (0.331)^∗^	0.256 (0.323)
Condition	-0.546 (0.329)^†^	-0.579 (0.314)^†^
Age × Condition	-0.583 (0.305)^†^	-0.783 (0.293)^∗∗^
Observations *(n)*	176	174


*^†^*p* < 0.10, ^∗^*p* < 0.05, ^∗∗^*p* < 0.01.*

*Logistic regression coefficients are the natural log (ln) of odd ratios for each predictor. Standard errors are presented in parentheses.*

Turning to the individual predictors, children in the Consensus condition were nearly twice as likely to endorse the novel-dance Protagonist’s label for the novel object as those in the Repetition condition (logistic regression coefficient = -0.579, *p* = 0.065, OR = 0.56 for the same-dance Protagonist). Age was a marginally significant predictor, such that older children were 1.4 times more likely to prefer the same-dance informant (logistic regression coefficient = 0.334, *p* = 0.076, OR = 1.397). However, a significant Condition by Age interaction indicates that with every year increase in age, children in the Consensus condition were 2.1 times as likely to endorse the novel-dance Protagonist’s label for the unfamiliar object, compared to the Repetition condition (logistic regression coefficient = -0.783, *p* = 0.008, OR = 0.457 for the same-dance Protagonist). Unlike the preference measure, sex was not a significant covariate for which Protagonist’s label children endorsed. This issue will be revisited in the general discussion.

#### Liking Predicts Learning

Children’s liking for a Protagonist significantly predicted whom they wanted to learn from. In a separate logistic regression model using Liking to predict informant choice, children who reported liking a Protagonist were five times as likely to learn from that same Protagonist than were children who did not report the same preference (logistic regression coefficient = 1.605, *p* < 0.001; OR = 4.98). In this model, Condition moderated by Age continues to be a significant predictor (logistic regression coefficient = -0.642, *p* = 0.039, OR = 0.526). That is, children increasingly differentiated their preferences across study conditions with age, showing a preference to learn from the novel-dance informant in the Consensus condition, and no clear preference in the Repetition condition. In this analysis, we removed Sex as a covariate, since it was a non-significant predictor in the full model, and including it greatly hampers the model’s predictions fit to the observed data. Hosmer and Lemeshow test indicate that the predicted data did not significantly differ from the observed data (χ^2^(8) = 5.594, *p* = 0.693), indicating good model fit. Together, preference (same-dance Protagonist, novel-dance Protagonist), condition, age, and an age-by-condition interaction term accounted for 22.8% of variability in children’s informant choices (Nagelkerke *R*^2^= 0.228) and also improved predictions of those choices on 19% of cases. Logistic Regression coefficients and standard errors for each predictor variable are shown in **Table 4**.

**Table 4:** Learning predicted by Liking, Age, Condition, and Age × Condition interaction term.

Predictors	Learning (*SE*)
Age (centered)	0.339 (0.202)^†^
Condition	-0.441 (0.340)
Age × Condition	-0.642 (0.310)^∗^
Liking	1.605 (0.362)^∗∗∗^
Observations *(n)*	169

*^†^*p* < 0.10, ^∗^*p* < 0.05, ^∗∗^*p* < 0.01, ^∗∗∗^*p* < 0.001.*

*Logistic regression coefficients are the natural log (ln) of odd ratios for each predictor. Standard errors are presented in parentheses.*

### Discussion

In both liking and learning measures, children’s choices differed by age. The youngest tested groups (2 and 3 year olds) did not differ in their choice of Protagonist across Consensus and Repetition conditions – it appears that they were insensitive to the distribution of information across individuals in our paradigm. In contrast, 4 and 5 year olds were influenced by behavioral consensus across individuals (they preferred the Protagonist who did a novel-dance), but not repetitive actions by a single individual (in which they chose the two Protagonists equally); this effect was more pronounced in older children, suggesting a greater readiness to discriminate individuals based on conventionality. The transitional age at which children in our sample differentiated between Consensus and Repetition conditions occurs around 4 years of age for preference, and a year later for informant choice, hinting at the possibility that preference informs model choice in this paradigm. Five-year-olds in our sample preferentially learned a novel object label from the novel-dance Protagonist, but were equally likely to learn from the same-dance Protagonist and novel-dance Protagonist in the Repetition condition, continuing the trajectory that emerges nearly a year earlier.

While these results suggest that the ability to differentiate between familiar and conventional information emerges around 4 years of age, we cannot rule out the possibility that they are due to age-related changes in domain-general processes, such as working memory. Indeed, as with all studies that report a developmental difference and an absence of a given ability at a young age, it is important to differentiate between children’s ability to perform on the task and their conceptual understanding. It is possible that two- and 3-year-old children’s results may be an artifact of immature memory for actors’ dances, rather than indifference between familiarity and conventionality *per se* (see Hamlin, 2014). Future research could explore whether age related differences in working memory accounts for the developmental findings we observed.

## General Discussion

We set out to examine whether preschool-aged children differentiate between conventional behaviors, performed by several members of a group, and equally frequent behaviors performed by just one member of a group. We demonstrated preschool-aged children were more likely to exhibit a social preference in the face of consensus behavior than frequent behavior. Furthermore, contrary to our initial hypotheses, children preferred to learn from individuals who performed novel actions versus those who performed conventional actions.

Children’s preferences for the unconventional actor indicate that they sometimes prefer innovative members of the group. While inconsistent with previous findings that children trust informants who were part of a consensus over a dissenter (Corriveau et al., 2009), these results are consistent with studies showing that children and adults are willing to learn from minorities who are successful (Scofield et al., 2013; Schillaci and Kelemen, 2014; Wilks et al., 2015). They are also consistent with models of cultural evolution, wherein occasional injection of innovations (through individual learning, or errors in social learning) to a cumulative repertoire help human groups adapt to changing environments (Lehmann et al., 2010; Boyd et al., 2011). Indeed, individuals who always produce behaviors that the rest of the group performs are necessarily limited as sources of new insights; thus, another reason to follow minorities may be to acquire innovative behaviors that the group does not yet know. This motivation may have driven children’s preferences and learning behaviors in the current studies (Legare and Nielsen, 2015).

Another (non-mutually exclusive) possibility for the disagreement between these findings and studies showing children prefer to learn from consensus members is the study’s methodological design. Previous research with 3-year-olds suggests that imitative fidelity is higher after witnessing synchronous than successive actors (Herrmann et al., 2013), presumably because synchronicity is a cue by which viewers infer that an act is a ritual. In this study, Demonstrators were shown to perform dances sequentially, rather than synchronously, and thus may have not cued the interpretation that the dances are performances of a ritual. Future studies may wish to examine adding ritual cues and their effects on children’s preferences for conventional models.

Another way in which our methodology may have produced disagreement with previous studies is that our study established consensus in one domain (dancing), and examined learning in a different domain (object labeling). Thus, children were initially introduced to the informants in a context where learning may not have been a relevant objective. Children’s subsequent desire to learn from a model may be informed by positive feelings toward the individual formed during the dance phase, rather than a direct assessment of their skill in word labeling. Future studies should attempt to tease apart these possibilities. The age patterns in our results provide some support for children’s choices being motivated by liking: 4 year olds in our sample reliably showed a preference for novel-dance Protagonists, a full year before they as a group reliably *learned* from novel-dance Protagonists. The timing of these effects, together with the strong relationship between children’s expressed preference and their subsequent choice of informant, suggests that children may first form a favorable impression of a Protagonist, which eventually informs who they choose to learn from in a different context. If so, it is possible that at least some proportion of children’s model choices are driven by a halo effect, whereby children simply learn from those they like, rather than any critical evaluation of potential models in each context a new (Dunham et al., 2011; Baron and Dunham, 2015). Previous studies’ reliance on single task measures may risk inflating the degree to which preschoolers demonstrate epistemic vigilance, especially in the context of longstanding relationships in which they like all the informants. Indeed, spillover effects in children’s informant choice have been observed to a limited extent in past studies (e.g., Chudek et al., 2012). Future studies may benefit from using more multi-task measures to explore the boundary conditions on such cross-task spillover.

An additional possibility is that children preferred and learned from the novel-dance Protagonist because they were relatively certain that all the individuals were part of the same group. That is, in previous studies where children have selectively learned from members of a consensus, group status has either not been made explicit, or it was clear that both in-group and out-group members were involved (Corriveau et al., 2009; Chen et al., 2013). In these situations, children may have used consensus behavior as a cue to who was in the same group, and preferred to learn from in-group members. In contrast, in the present paper all characters were Smurfs, they were introduced together, and the study was run just after a Smurfs movie was released that many participants reported seeing. For these reasons, presumably children believed that all the characters were part of the same “Smurfs” group. If so, children may not have needed to use conventionality as a cue to group membership or group-specific knowledge, and so were free to evaluate Protagonists’ behaviors based on other factors, such as creativity or added informational value.

A further possibility is that children’s preferences could have been driven by a desire for identity expression. In our particular experimental set-up, children were invited to play a game, and likely assessed it to be a situation in which uniqueness and self-expression are acceptable. Indeed, these qualities are often encouraged by the broader North American culture (Markus and Kitayama, 1991; Bond and Smith, 1996). Furthermore, there were no obvious repercussions for learning from the “wrong” model in our paradigm, making our results consistent with previous work suggesting that people’s reliance on conformity decreases as the stakes of accuracy decrease (Baron et al., 1996). Children in our study may have perceived the learning task as having low-stakes, and therefore saw it as an opportunity more suited to expressing their individuality than to accurately learning an object label.

Two patterns of results in the current studies are suggestive that the experimental paradigm may have cued social contexts where self-expression (versus adhering to social convention) is normative and appropriate: (1) older children showed stronger preferences for the unconventional actor, since greater levels of acculturation occurs with age, and (2) boys showed a stronger preference for the unconventional actor than girls in the older age group, as females are more likely to receive stronger cultural pressures to conform (e.g., Block, 1973; Hansson et al., 1980; Eagly, 1983; Eagly and Wood, 1985). This possibility warrants further research into how the importance of accuracy of learning outcomes (i.e., stakes) affect children’s choices to learn from conventional vs. unconventional individuals.

Children’s selective social preferences based on prevalent behaviors suggest that at an early age, humans are sensitive to group-relevant behaviors, independent of familiarity. Whether or not one adheres to group conventions increasingly inform preschool aged children’s choice of social partners, and cultural models. Children’s use of consensus information appear to lead to context-dependent preferences, suggestive of competing motives to adhere to group conventions and to acquire new information. Overall, these studies point to an early ontogeny of group level reasoning that aid young humans in learning about social conventional knowledge.

## Author Contributions

WZ, AB, and JH designed the study, WZ carried out data collection and statistical analyses, WZ wrote the manuscript with input from AB and JH. All authors granted final approval of the manuscript version to be published.

## Conflict of Interest Statement

The authors declare that the research was conducted in the absence of any commercial or financial relationships that could be construed as a potential conflict of interest.
